# Assessment of Knowledge and Perception Regarding Deep Brain Stimulation Among Medical Students in Saudi Arabia

**DOI:** 10.7759/cureus.41540

**Published:** 2023-07-07

**Authors:** Sarah S Aldharman, Fadi A Munhish, Haila A Alabssi, Maryam A Alamer, Fay A Althunayyan, Majidah H Halawi, Shireen H Elfaham, Taghreed A Alsinani, Saud A Alnaaim

**Affiliations:** 1 College of Medicine, King Saud Bin Abdulaziz University for Health Sciences, Riyadh, SAU; 2 College of Medicine, Jazan University, Jazan, SAU; 3 College of Medicine, Imam Abdulrahman Bin Faisal University, Dammam, SAU; 4 College of Medicine, King Faisal University, Al-Hofuf, SAU; 5 College of Medicine, Ibn Sina National College for Medical Studies, Jeddah, SAU; 6 Department of Surgery, King Fahad General Hospital, Jeddah, SAU; 7 Department of Clinical Neurosciences, King Faisal University, Al-Hofuf, SAU

**Keywords:** saudi arabia, neurology, medical students, deep brain stimulation, education, knowledge

## Abstract

Background: Deep brain stimulation (DBS) is a neurosurgical procedure approved for treating psychiatric and movement disorders, including Parkinson's disease (PD), essential tremor, dystonia, and other neurological conditions. The widespread use of DBS may not be reflected in the medical education curricula in Saudi universities, thus jeopardizing future patients' access to it. This study aims to investigate the knowledge and attitudes of medical students toward DBS as a therapeutic option.

Method: A descriptive cross-sectional questionnaire-based study was conducted. The survey was distributed on online platforms to acquire responses from different regions of Saudi Arabia. The target population was medical students in the preclinical and clinical phases of medical education from different regions of Saudi Arabia.

Results: A total of 1075 medical students from various medical schools in Saudi Arabia were included. More than half of the students aged 21 to 23 (50.1%) were females (63.2%). More than half of the students have correctly recognized DBS as a Food and Drug Administration (FDA)-approved treatment (59.7%). Only 20.1% of the students stated that they received adequate education/training about DBS. About 53.8% of the students had self-rated their knowledge as poor, whereas 20.6% had rated their knowledge as good. A negative bias was more observed among the older students and students with a family history of DBS treatment. Half of the participants (54.1%) indicated that DBS is associated with severe adverse effects. A significant association between the level of knowledge about DBS and the academic level was observed.

Conclusion: Almost half of the medical students had poor knowledge and unfavorable attitude toward DBS in Saudi Arabia. The current medical curricula are incommensurable with the clinical implications of DBS, which may deny future patients from such an effective therapeutic option. We recommend incorporating DBS teaching sessions to enhance future physicians' awareness and understanding of the benefits of this intervention.

## Introduction

Deep brain stimulation (DBS) is a neurosurgical procedure that uses implanted electrodes and electrical stimulations to treat movement disorders among other neurological conditions. It is approved by the United States Food and Drug Administration (FDA) for treating Parkinson's disease (PD), essential tremor, dystonia, and other neurological conditions [1,2]. When there is an activity imbalance in electrical signals, DBS is used to stimulate specific areas in the brain to help treat movement-related symptoms [1]. Recently, brain disorders accounted for more than a quarter of all health loss resulting from disabilities. PD is associated with a twofold increase in the risk of death with a significant impact on mobility and balance [3].

DBS has become one of the significant improvements in surgical techniques. DBS became the treatment of choice for patients with pharmacoresistant motor fluctuations in PD and has completely replaced the need for ablative surgery in the treatment of advanced PD, leading to better results, less morbidity, and lower mortality [4]. DBS substantially impacts how neurological disorders are treated and the investigation of the neural populations and pathways involved in their pathologies with new tools, such as optogenetics [5].

Another study was conducted in 2021 at Virginia Tech Carilion School of Medicine on a sample size of 65 medical students, 49% of whom were in the preclinical phase (years 1 and 2) and 51% were in clinical training (years 3 and 4) [6]. The students were analyzed separately to assess their attitudes and knowledge changes with clinical exposure to DBS [6]. The study found that 65% of the students believed that they were not well educated about DBS, 10.6% believed that DBS was associated with severe adverse effects, and 36% of the students were unsure of the FDA approval of the DBS therapy [6]. The study concluded that the overall attitude of students was positive, and there was no difference between the preclinical- and clinical-year students, which could be attributed to the lack of exposure during the clinical years of medical education [6].

In another study, Prasad et al. indicated significant gaps in the knowledge and awareness of DBS among patients with PD and their caregivers [7]. They suggested that adequate education is necessary to clarify any misconceptions about DBS and avoid poor satisfaction and unrealistic expectations [7]. Furthermore, Wloch et al. reported that medical students’ knowledge of DBS was limited before entering the medical practice and that there is a moderate gain in knowledge over the following few years regarding common disorders, such as PD and tremors, which are known indications of DBS during education [8]. However, knowledge about the impact of DBS on specific symptoms in PD and about DBS targets was limited in both groups [8].

There is a scarcity of literature regarding medical students' knowledge and perception of DBS in Saudi Arabia. Therefore, the purpose of this study was to fill the gap in the literature for a better understanding of this topic. This study also aimed to assess medical students' knowledge and perception of DBS as a treatment modality in Saudi Arabia.

## Materials and methods

This is a descriptive cross-sectional questionnaire-based study. Data were collected through an online self-administered questionnaire. The survey was distributed on online platforms to acquire responses from different regions of Saudi Arabia. The study was carried out from September 2022 to April 2023.

Study population

Our targeted population was medical students in the preclinical and clinical phases of medical education from different regions of Saudi Arabia (central, southern, eastern, western, and northern). The number of medical colleges of universities is approximately 30 in Saudi Arabia [9].

Sample size and sampling technique

OpenEpi® version 3.0 software was used to estimate the sample size. The representative sample size required was 384, with the margin error determined as 5% and the confidence level determined as 95%. We aimed to gather more than the estimated sample size to overcome any exclusions. Non-probability convenience sampling techniques were utilized.

Inclusion and exclusion criteria

The eligibility criteria included both genders, aged at least 18 years old, and being a medical student in Saudi Arabia. Any participant who did not agree to participate or did not complete the questionnaire was excluded from the analysis.

Data collection tools

The questionnaire was adapted from a previous study [6]. It contained 24 items, with the first five items seeking demographic data of the participants (including age, gender, region, year in medical school, intended specialty, and any history of a family member treated with DBS). The remaining sections are a nine-item knowledge inventory, a seven-item bias inventory to assess attitudes toward DBS, and a self-assessment of knowledge question. Furthermore, one independent item, self-perceived education on DBS, evaluated whether the participant believed that they had received adequate training about DBS and its therapeutic uses. Questions addressed particular indications, procedures, side effects, effects on movement and psychiatric symptoms, and personal attitudes toward treating or suggesting a relative to be treated with DBS.

The knowledge inventory only allowed three replies: “Yes” (+1), “No” (1), and “I do not know” (0). The bias inventory was graded on a five-point Likert scale, ranging from “strongly agree” to “strongly disagree.” Each participant received a knowledge inventory score and a bias inventory score after the scores for each inventory were individually added. Self-assessment of knowledge was evaluated by asking the participants to rate their understanding of DBS on a seven-point scale, ranging from 1 = vague understanding to 7 = thorough understanding.

The first page was designated for informed consent. The questionnaire was provided electronically using Google Forms (Google LLC, Mountain View, California, United States). It was distributed on social media platforms, such as WhatsApp, Twitter, and Telegram. After completing the questionnaires, they were checked for completeness and any missing information. The online data gathering system ensured that all items must be answered, ensuring that no incomplete forms were submitted. Then, the data were entered and coded in Microsoft Excel. All information was confidential, and participation in this research is voluntary and optional. The data analysis and publication process did not require any identifiable personal data. The study was conducted in agreement with the principles of the Declaration of Helsinki, and all participants were informed of the nature and objectives of the study at the beginning of the survey. The approval for the study was obtained from the Research Ethics Committee at King Faisal University in Al Hofuf, Saudi Arabia, with approval no. KFU-REC-2023-FEB-ETHICS646.

Coding and scoring

In the current study, we relied on nine items to assess students' knowledge regarding DBS. Of these, the responses of eight items were coded as “No” = -1, “I do not know” = 0, and “Yes” = 1. For one item that indicates the impact of DBS on worsening or improving the psychiatric illness, the responses were coded as "Worsen" = -1, "I do not know" = 0, and "Improve" = 1. A knowledge score was computed by summing up the correct responses, and higher scores indicated a higher level of knowledge.

For the bias domain, we incorporated seven relevant items. Two had positive statements, namely, "I would advise a close relative to receive DBS if recommended" and "If required, I would undergo DBS." By contrast, the remaining items indicated a negative bias. The responses to these items were collected on a five-point Likert scale, ranging from strongly disagree = -1, disagree = -0.5, neither disagree nor agree = 0, agree = 0.5, and strongly agree = 1. The positively worded statements were reverse-coded. Consequently, a biased score was computed by summing up the item values, such that a higher score indicated a negative bias.

Regarding the self-perception of the knowledge domain, the students rated their self-knowledge on a seven-point scale from “poor understanding” = 1 to “thorough understanding” = 7. The item was analyzed as a numerical variable, and a histogram was developed to express the frequency distribution of the self-perceived knowledge.

Statistical analysis

Statistical analysis was conducted using RStudio software (R version 4.1.1). We expressed categorical variables as frequencies and percentages. To assess the differences between the students' demographic groups in terms of their different scores, we constructed three multivariate linear regression models using the scores of knowledge, negative bias, and having a self-perception of good knowledge as dependent variables in each model. The following demographic characteristics were entered as independent variables: age, gender, region, academic year, intended specialty, and having a family member that has been treated with DBS. These independent variables were entered into the models using a stepwise forward selection method. The regression analysis results were presented as beta coefficients and their respective 95% confidence intervals (95% CIs). A p-value of <0.05 indicates statistical significance.

## Results

Sociodemographic characteristics

**Figure 1:**
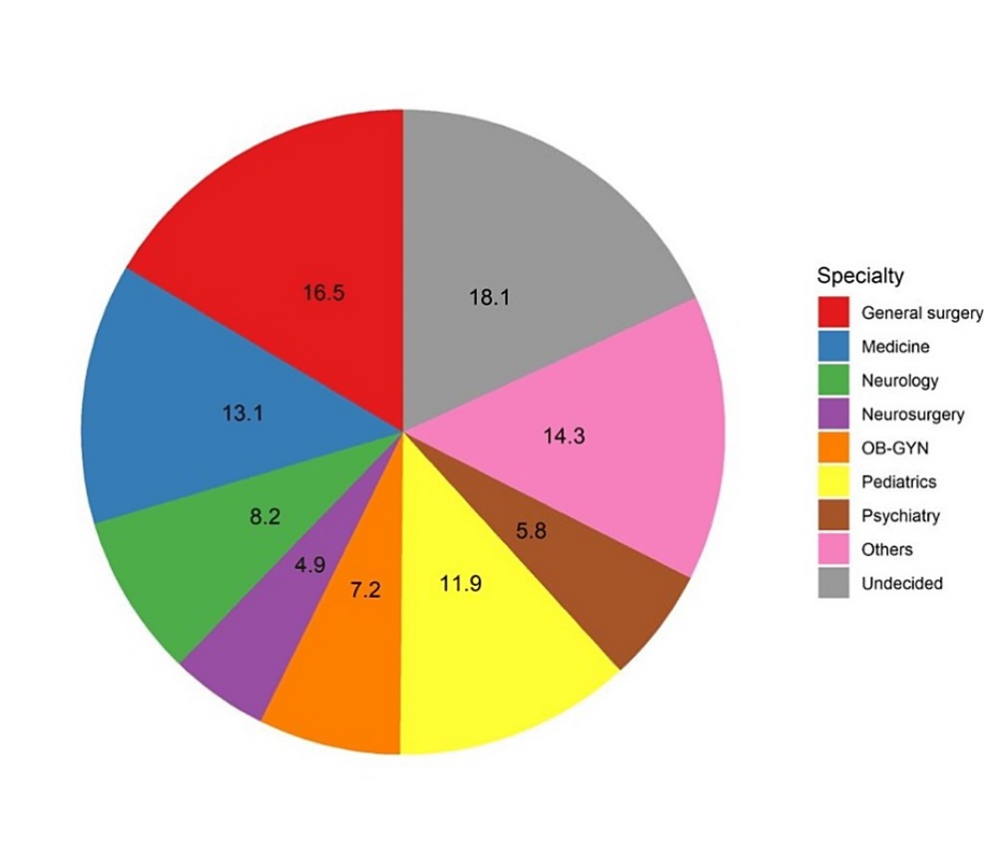
Students’ responses regarding their intended specialties.

Initially, we received 1175 responses on the online platform. We excluded nine responses from those who declined to participate, 51 responses from non-medical students, and 40 responses from those who were aged <18 years. Consequently, the responses of the 1075 participants were analyzed. More than half of the students aged 21 to 23 (50.1%) were females (63.2%). Almost one-quarter of the participants were residing in the central region of Saudi Arabia (24.7%), whereas only 9.8% were residing in the northern region. Most students were studying in the clinical phase of medical education (77.9%). Notably, 12.8% of the respondents had a family member treated with DBS (Table 1). A considerable proportion of the students were undecided about their intended specialty (18.1%), while 16.5% and 13.1% intend to specialize in general surgery and medicine, respectively (Figure 1).

**Table 1:** : Sociodemographic characteristics. DBS: deep brain stimulation

Parameter	Category	N (%)
Age (years)	≤20	147 (13.7%)
	21 to 23	539 (50.1%)
	>23	389 (36.2%)
Gender	Male	396 (36.8%)
	Female	679 (63.2%)
Region	Central	266 (24.7%)
	Western	222 (20.7%)
	Eastern	262 (24.4%)
	Northern	105 (9.8%)
	Southern	220 (20.5%)
Year	Preclinical phase	238 (22.1%)
	Clinical phase	837 (77.9%)
Have a family member that has been treated with DBS	Yes	138 (12.8%)

Knowledge regarding DBS and self-perception of education/training

**Figure 2:**
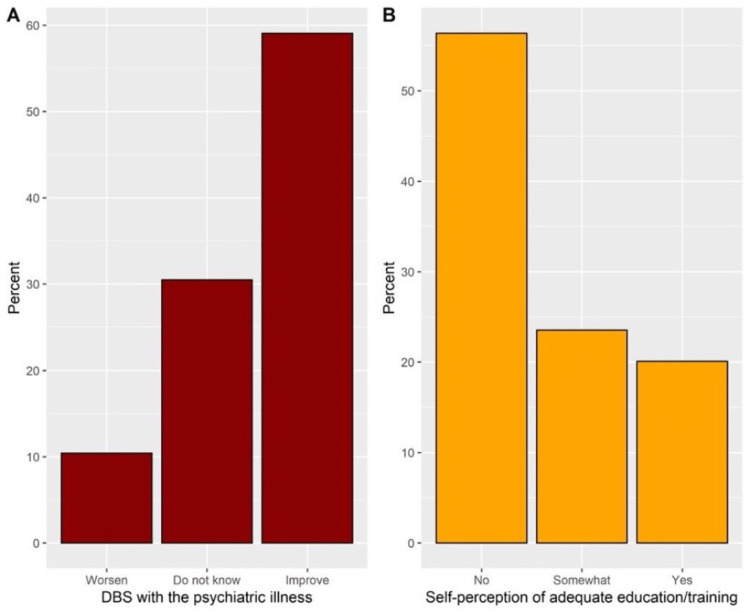
Students’ responses to the impact of DBS on a psychiatric illness (A) and their perceptions regarding receiving adequate education or training about DBS (B).

**Figure 3:**
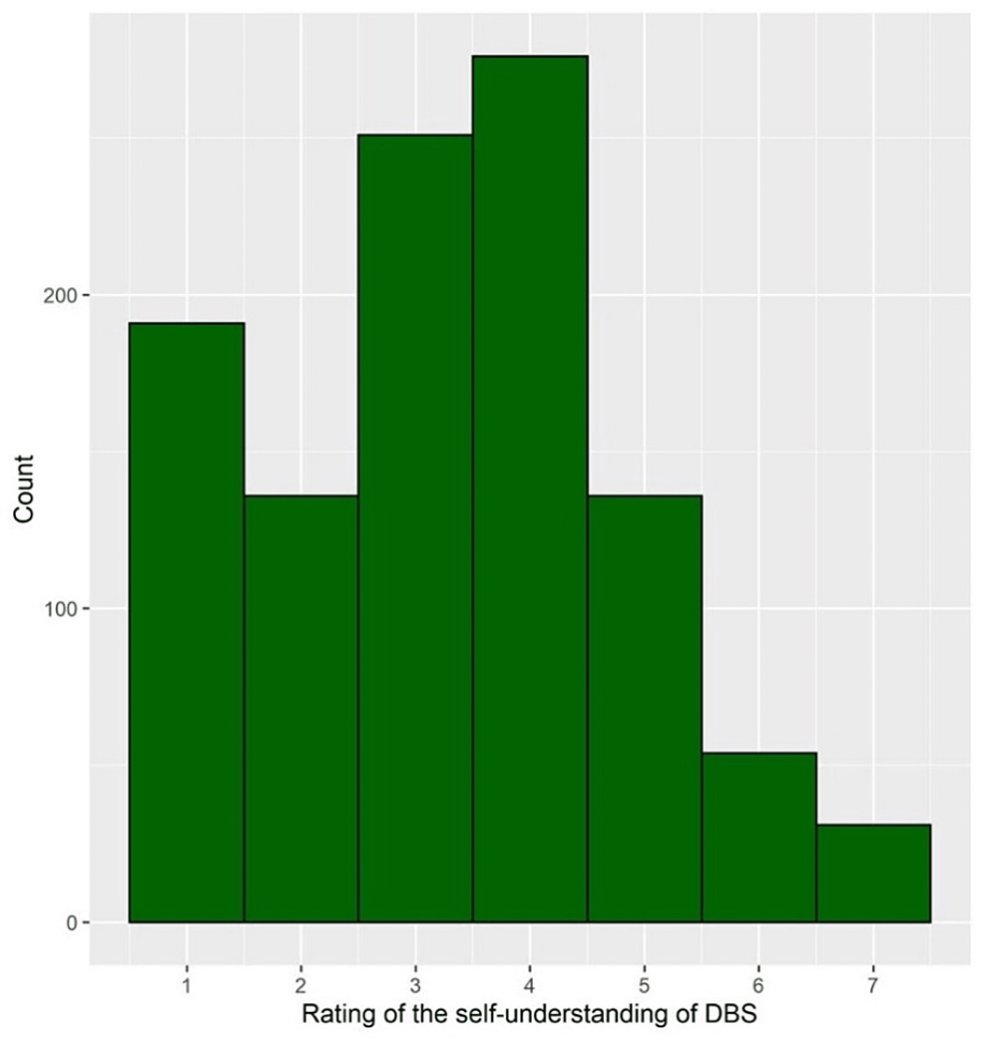
Histogram depicting the frequency distribution of self-reported knowledge regarding DBS.

In general, more than half of the students have correctly recognized DBS as an FDA-approved treatment (59.7%), DSB as a helpful approach in treating psychiatric disorders (57.3%), and the involvement of neurologists in DBS administration (56.6%). On the other hand, 29.9% of the students did not correctly identify that DBS effects last only a short while, and 26.9% of them incorrectly stated that DBS does not result in a permanent cure (Table 2). In addition, more than half of the students (59.1%) have correctly indicated that DBS improves psychiatric illnesses (Figure 2A). Notably, only 20.1% of the students declared that they received adequate education or training about DBS (Figure 2B). Concerning the self-rated knowledge about DBS, the results revealed that 53.8% of the students self-rated their knowledge from 1 to 3 (poor), whereas 20.6% of them self-rated their knowledge from 5 to 7 (good; Figure 3).

**Table 2:** Students’ responses to eight knowledge items. DBS: deep brain stimulation; FDA: Food and Drug Administration

Parameter	No	Yes	I do not know
Is DBS an FDA-approved treatment?	65 (6.0%)	642 (59.7%)	368 (34.2%)
Is DBS useful in treating psychiatric disorders?	183 (17.0%)	616 (57.3%)	276 (25.7%)
Is DBS useful in treating movement disorders?	190 (17.7%)	533 (49.6%)	352 (32.7%)
Do the effects of DBS last only a short while?	321 (29.9%)	285 (26.5%)	469 (43.6%)
Does DBS result in a permanent cure?	289 (26.9%)	318 (29.6%)	468 (43.5%)
Are psychiatrists involved in administering DBS?	184 (17.1%)	536 (49.9%)	355 (33.0%)
Are neurologists involved in administering DBS?	176 (16.4%)	608 (56.6%)	291 (27.1%)
Are neurosurgeons involved in administering DBS?	192 (17.9%)	532 (49.5%)	351 (32.7%)

Bias toward DBS

Regarding the bias-related items, a considerable proportion of the students agreed or strongly agreed that DBS is associated with severe adverse effects (54.1%) and that students would undergo DBS if indicated (52.6%). Conversely, more than a quarter of the students disagreed or strongly disagreed that DBS is often given to people who do not need it (28.9%) and that DBS is dangerous and should not be used (27.7%; Table 3).

**Table 3:** Students’ responses to bias-related items.

Parameter	Strongly disagree	Disagree	Neither disagree or agree	Agree	Strongly agree
The procedure is associated with severe adverse effects.	20 (1.9%)	102 (9.5%)	371 (34.5%)	210 (19.5%)	372 (34.6%)
The procedure is associated with brain damage.	41 (3.8%)	182 (16.9%)	410 (38.1%)	283 (26.3%)	159 (14.8%)
DBS is a painful procedure.	50 (4.7%)	153 (14.2%)	426 (39.6%)	275 (25.6%)	171 (15.9%)
DBS is dangerous and should not be used.	72 (6.7%)	226 (21.0%)	417 (38.8%)	214 (19.9%)	146 (13.6%)
I would advise a close relative to receive DBS if recommended.	44 (4.1%)	66 (6.1%)	411 (38.2%)	368 (34.2%)	186 (17.3%)
If required, I would undergo DBS.	42 (3.9%)	67 (6.2%)	401 (37.3%)	372 (34.6%)	193 (18.0%)
DBS is often given to people who do not need it.	141 (13.1%)	170 (15.8%)	387 (36.0%)	219 (20.4%)	158 (14.7%)

Predictors of students’ knowledge

Considering the students residing in the central region as a reference group, residents of other geographical regions had independently low knowledge scores, including those from the western (beta = -1.45; 95% CI = -2.01 to -0.90; p < 0.001), eastern (beta = -1.16; 95% CI = -1.69 to -0.63; p < 0.001), northern (beta = -0.93; 95% CI = -1.63 to -0.23; p = 0.009), and southern regions (beta = -1.12; 95% CI = -1.68 to -0.57; p < 0.001). In addition, the following student groups were more likely to have low knowledge scores: students who intended to join other specialties (beta = -0.74; 95% CI = -1.39 to -0.09; p = 0.025) and those who had a family member who received DBS (beta = -0.59; 95% CI = -1.14 to -0.04; p = 0.035; Table 4).

**Table 4:** Results of the regression analysis for the predictors of students’ knowledge, negative bias, and having a self-perception of good knowledge. CI: confidence interval; DBS: deep brain simulation

Parameter	Category	Knowledge		Negative bias		Self-perception of good knowledge	
		Beta (95% CI)	p-value	Beta (95% CI)	p-value	Beta (95% CI)	p-value
Age (years)	<=20	—		—		—	
	21 to 23	0.16 (-0.52 to 0.83)	0.654	-0.09 (-0.52 to 0.33)	0.663	0.52 (0.20 to 0.84)	0.002
	>23	-0.04 (-0.78 to 0.69)	0.906	0.58 (0.12 to 1.05)	0.013	0.97 (0.62 to 1.32)	<0.001
Gender	Male	—		—		—	
	Female	-0.10 (-0.49 to 0.30)	0.639	-0.01 (-0.26 to 0.24)	0.935	-0.16 (-0.35 to 0.03)	0.094
Region	Central	—		—		—	
	Western	-1.45 (-2.01 to -0.90)	<0.001	-0.04 (-0.39 to 0.31)	0.832	-0.08 (-0.35 to 0.18)	0.544
	Eastern	-1.16 (-1.69 to -0.63)	<0.001	0.11 (-0.22 to 0.44)	0.516	0.33 (0.07 to 0.58)	0.011
	Northern	-0.93 (-1.63 to -0.23)	0.009	-0.20 (-0.64 to 0.23)	0.360	0.88 (0.55 to 1.21)	<0.001
	Southern	-1.12 (-1.68 to -0.57)	<0.001	0.29 (-0.06 to 0.64)	0.104	0.15 (-0.12 to 0.41)	0.274
Year	Preclinical phase	—		—		—	
	Clinical phase	0.05 (-0.51 to 0.61)	0.869	-0.44 (-0.79 to -0.09)	0.014	-0.54 (-0.80 to -0.27)	<0.001
General surgery	No	—		—		—	
	Yes	-0.29 (-0.92 to 0.33)	0.358	0.59 (0.20 to 0.99)	0.003	0.29 (-0.01 to 0.59)	0.054
Medicine	No	—		—		—	
	Yes	-0.43 (-1.09 to 0.23)	0.201	0.27 (-0.14 to 0.68)	0.200	0.48 (0.17 to 0.80)	0.003
Neurology	No	—		—		—	
	Yes	0.17 (-0.61 to 0.95)	0.671	-0.03 (-0.52 to 0.45)	0.892	0.72 (0.35 to 1.09)	<0.001
Neurosurgery	No	—		—		—	
	Yes	-0.63 (-1.55 to 0.29)	0.182	0.21 (-0.37 to 0.78)	0.481	0.63 (0.19 to 1.07)	0.005
Obstetrics and gynecology	No	—		—		—	
	Yes	-0.05 (-0.86 to 0.76)	0.908	0.69 (0.18 to 1.19)	0.008	0.65 (0.26 to 1.04)	0.001
Pediatrics	No	—		—		—	
	Yes	-0.57 (-1.27 to 0.12)	0.104	0.87 (0.44 to 1.31)	<0.001	0.44 (0.11 to 0.77)	0.009
Psychiatry	No	—		—		—	
	Yes	0.01 (-0.87 to 0.88)	0.987	0.57 (0.02 to 1.12)	0.042	0.76 (0.34 to 1.18)	<0.001
Others	No	—		—		—	
	Yes	-0.74 (-1.39 to -0.09)	0.025	-0.03 (-0.44 to 0.37)	0.867	0.08 (-0.23 to 0.38)	0.632
Have a family member that has been treated with DBS	No	—		—		—	
	Yes	-0.59 (-1.14 to -0.04)	0.035	0.52 (0.17 to 0.86)	0.003	1.01 (0.75 to 1.28)	<0.001

Predictors of negative bias toward DBS

A negative bias was more likely to be apparent among the older students (beta = 0.58; 95% CI = 0.12 to 1.05; p = 0.013) and students who had a family history of DBS treatment (beta = 0.52; 95% CI = 0.17 to 0.86; p = 0.003), as well as students who intended to be allocated to the following specialties: general surgery (beta = 0.59; 95% CI = 0.20 to 0.99; p = 0.003), obstetrics and gynecology (beta = 0.69; 95% CI = 0.18 to 1.19; p = 0.008), pediatrics (beta = 0.87; 95% CI = 0.44 to 1.31; p < 0.001), and psychiatry (beta = 0.57; 95% CI = 0.02 to 1.12; p = 0.042). By contrast, the students of the clinical phase were less likely to demonstrate a negative bias toward DBS (beta = -0.44; 95% CI = -0.79 to -0.09; p = 0.014; Table 4).

Predictors of self-perception of good knowledge

The students of the clinical phase had a lower likelihood of possessing poor self-perception of knowledge (beta = -0.54; 95% CI = -0.80 to -0.27; p < 0.001). However, self-perceived knowledge was independently associated with more advanced ages (beta = 0.52, 95% CI = 0.20 to 0.84, and p = 0.002 for students aged 21 to 23 years and beta = 0.97, 95% CI = 0.62 to 1.32, and p < 0.001 for students aged >23 years), having a family member who received DBS (beta = 1.01; 95% CI = 0.75 to 1.28; p < 0.001), and residing in the eastern and northern regions (beta = 0.33, 95% CI = 0.07 to 0.58, p = 0.011 and beta = 0.88, 95% CI = 0.55 to 1.21, p < 0.001, respectively). Furthermore, the students who intended to undertake the following specialties had significantly higher self-perceived knowledge: medicine (beta = 0.48; 95% CI = 0.17 to 0.80; p = 0.003), neurology (beta = 0.72; 95% CI = 0.35 to 1.09; p < 0.001), neurosurgery (beta = 0.36; 95% CI = 0.19 to 1.07; p = 0.005), obstetrics and gynecology (beta = 0.65; 95% CI = 0.26 to 1.04; p = 0.001), pediatrics (beta = 0.44; 95% CI = 0.11 to 0.77; p = 0.009), and psychiatry (beta = 0.76; 95% CI = 0.34 to 1.18; p < 0.001; Table 4).

## Discussion

This study aimed to explore the current knowledge and perception of medical students in Saudi Arabia toward DBS. Our findings demonstrate that, among the polled students, there is a primarily good knowledge of DBS, which is expected as the majority of the students were in the clinical phase of medical education (77.9%) and 12.8% of the respondents had a family member who has been treated with DBS. Furthermore, previous research has linked the correlation between the increase in clinical exposure with the increased fund of knowledge and clinical confidence about DBS [6]. Our results show that more than half of the students appreciated DBS as a valuable approach to treating psychiatric disorders (57.3%). In a previous study of surveyed students, 36% were unsure of the FDA approval status of DBS treatment [6].

Nevertheless, a troubling finding in our study was that 29.9% of the students did not correctly identify that DBS effects last only a short while, and 26.9% of them had incorrectly stated that DBS does not result in a permanent cure. These practical findings are justified by the small percentage (20.1%) of students who declared that they received adequate education or training about DBS. Meanwhile, it is concerning how inadequately they were trained in this therapeutic approach. Although psychiatry and neurology are major subjects in medical school, DBS is not adequately comprehended within the existing standard medical curriculum. This finding is similar to earlier studies that showed that medical students' ignorance of electroconvulsive therapy (ECT) led to biases toward the treatment modality [6,10].

A negative bias was more likely to be apparent among students who intended to be allocated to the following specialties: general surgery, obstetrics and gynecology, and psychiatry. Similarly, in a previous study, it was found that this bias is associated with studying neuroscience subjects, including psychiatry, neurology, and neurosurgery [11]. Our study revealed that students of the clinical phase were less likely to possess poor self-perception of knowledge. By contrast, there was no observed difference between the surveyed preclinical- and clinical-phase students in a previous study [6]. Meanwhile, a German study found that students gain a greater understanding of DBS as they progress throughout their senior year curriculum [8]. A previous study conducted by Kimmerle et al. found that it is unusual for laypeople to know DBS [12]. Interestingly, many physicians are unaware of DBS's current applications and clinical role in treating various neuropsychiatric conditions [13]. 

Our study has some limitations. First, the study's cross-sectional nature might limit the interpretation of any association revealed by this article. Associations from the cross-sectional investigation may be less definitive than other study types. Second, the student's responses may be affected by the recall bias. Finally, most students were studying in the clinical phases, which may affect the generalizability of the results. Convenient sampling and use of the authors' network, sampling bias, and the dominance of the female gender are a few other limitations of the study.

Assessing medical students' knowledge regarding DBS is essential regardless of their specialty of interest as they may encounter patients who have undergone or may need DBS procedure. Medical educators should consider incorporating DBS into existing neuroscience curricula at medical schools to fill the knowledge gap. We recommend further studies on assessing the knowledge of resident doctors from different specialties about DBS. Moreover, educational campaigns, medical webinars, and conferences should be held to increase the medical community's awareness of dealing with such a modality. Other studies may consider assessing factors influencing the knowledge of DBS in medical training. Furthermore, studies may apply to assess the misconceptions about DBS among the general public and its association with the delayed decision to be used.

## Conclusions

Our study revealed that almost half of the participants showed a poor level of self-perceived knowledge of DBS. Students in the clinical phase of medical education were less likely to have a negative bias toward DBS in comparison to older students or students with a family background of DBS treatment. About half of the students (54.1%) agreed that DBS is associated with severe adverse effects. To effectively serve patients who could benefit from DBS, medical students must get enough education that disproves common misconceptions and ensure adequate knowledge.
